# Impact of nasal modifications on sinonasal function after maxillomandibular advancement for obstructive sleep apnea

**DOI:** 10.1007/s11325-025-03262-x

**Published:** 2025-02-08

**Authors:** Nicolas S. Poupore, Mohamed Abdelwahab

**Affiliations:** Sleep and Facial Skeletal Surgery, Department of Otolaryngology– Head & Neck Surgery (O-HNS), Department of Oral and Maxillofacial Surgery, Division of Sleep Surgery, Medical University of South Carolina, 135 Rutledge Avenue, MSC 550, Charleston, South Carolina, SC 29425 USA

**Keywords:** Maxillomandibular advancement, Sinonasal function, Postoperative, Obstructive sleep apnea

## Abstract

**Purpose:**

To assess sinonasal function after preservation maxillomandibular advancement (MMA), as initial reports have shown it may decrease postoperatively.

**Methods:**

This prospective study was performed at a tertiary referral center starting January 2023. MMA was performed with previously published nasal modifications to help mitigate negative sinonasal outcomes. Sino-nasal Outcome Test (SNOT-22) and Nasal Obstruction Symptom Evaluation Survey (NOSE) were collected preoperatively, and one and three months postoperatively. Repeated measures ANOVAs with a Bonferroni adjustment were performed for total score, total sinonasal score (sum of questions 1–12), and each symptom. Both *p*-values and partial eta squared (n_p_^2^) were reported.

**Results:**

Twenty patients were included. Median age was 50.7 years (range 31–65), with 20.0% being female. Preoperative AHI (65.1 ± 28.9) and SpO2 Nadir (78.0% [69.0–82.0]) improved to 12.1 ± 12.1 and 86.0 ± 3.2%. NOSE significantly decreased at one month (55.9 ± 28.4 vs. 8.11 ± 12.0, *p* < 0.001). Total score and total sinonasal scores significantly decreased postoperatively (49.0 ± 21.6 vs. 18.1 ± 17.4 vs. 12.5 ± 14.1, *p* < 0.001; 17.3 ± 12.5 vs. 9.2 ± 9.3 vs. 6.3 ± 7.3, *p* = 0.003) with MMA having large effects on both variables (n_p_^2^=0.72 and 0.35, respectively). MMA had large significant effects on improvement in need to blow nose, nasal blockage, sneezing, runny nose, cough, post-nasal discharge, dizziness, and ear pain at one and three months postoperatively. Facial pain/pressure significantly worsened at one-month but then improved to baseline at three months postoperatively ((1.2 ± 1.4 vs. 1.9 ± 1.5 vs. 1.2 ± 1.4, *p* = 0.026).

**Conclusion:**

Patients who underwent preservation MMA did not show evidence of worsening sinonasal function, with some evidence that sinonasal function may improve after MMA at three months postoperatively. Long-term follow-up with more patients is needed to support these findings.

## Introduction

Maxillomandibular advancement (MMA) has consistently been shown to be an effective surgical intervention for the treatment of obstructive sleep apnea (OSA), particularly in patients with difficult-to-treat or severe OSA [1]. This orthognathic procedure produces its effects by stabilizing the upper airway soft tissue and musculature, specifically the tensor palatini, palatoglossus, and palatopharyngeus muscles [2–4]. These changes have been effectively visualized and quantified on postoperative drug-induced sleep endoscopy (DISE) and correlated with postoperative health-related and functional success after MMA [5, 6]. Furthermore, while research on long-term outcomes after MMA develops, initial results suggest long-term persistence of surgical success [5, 6].

One of the concerns or sequelae with MMA is the postoperative compromise in sinonasal function, with initial evidence showing that 19% of patients had postoperative nasal obstruction requiring corrective surgery [7]. In the orthognathic literature, it was shown that 20–70% of patients after Le Fort I experienced nasal obstruction [8]. Many different reasons have been proposed for reduction in sinonasal function, including buckling of the nasal septum, narrowing of the internal nasal valve, changes to the sinonasal mucosa, alteration of the osteomeatal complex, decrease in maxillary size, and increase in airway resistance [2, 7, 9, 10]. Furthermore, it has been shown that greater maxillary advancements were more harmful to the sinonasal complex [2]. This has become a challenge for providers performing MMA because while more maxillary advancement has been suggested to provide greater improvements in OSA parameters and sleep function, sinonasal function is crucial to improve OSA and improves one’s quality of sleep, therefore potentially dampening the effects of the maxillary advancement [2, 11]. To address this issue, advancements in improving sinonasal function after MMA by using a preservation and contemporary technique have been proposed by our team.

However, increasing rates of rhinosinusitis has not been thoroughly investigated in patients undergoing MMA. Therefore, we present a prospective, single-institution observational study using Sino-nasal Outcome Test (SNOT-22) to analyze the sinonasal function after MMA using the preservation technique described herein [2, 12, 13]. We hypothesize that these modifications will not worsen (or may optimize) patient’s sinonasal function after MMA.

## Materials and methods

### Patient selection

**Fig. 1:**
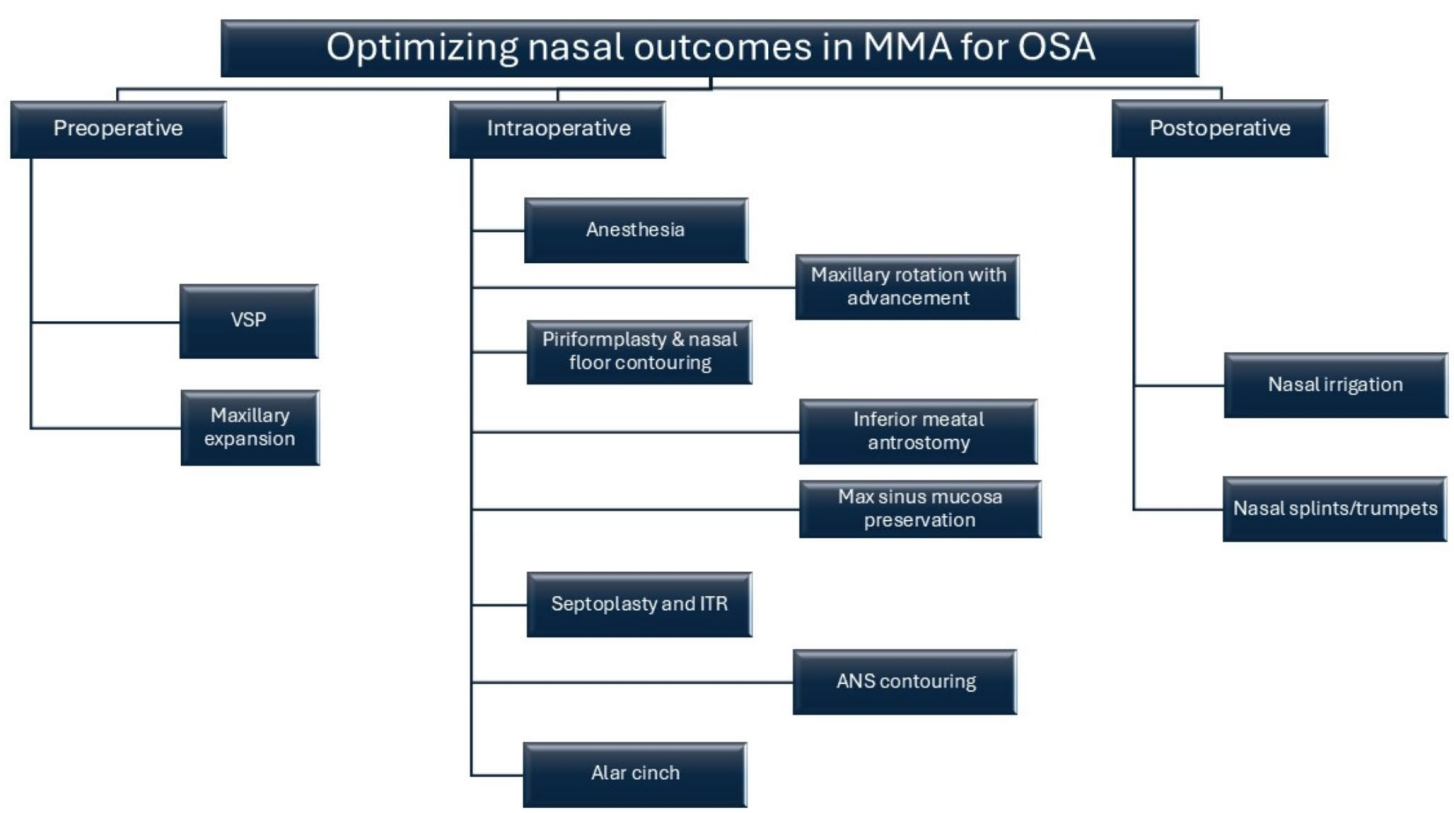
Optimizing nasal outcomes in MMA for OSA

Medical University of South Carolina’s Institutional Review Board for Human Research approval (Pro00129710) was obtained to collect prospective data on adults who underwent MMA at this tertiary institution. All patients were older than 18 years old with OSA confirmed by polysomnography (PSG). PSGs were either performed internally at our institution or externally. In-laboratory or home sleep studies were permitted for inclusion. There was no particular time frame for how recent a sleep study needed to be, but generally, a repeat PSG was performed if the sleep study was over two years old. All patients underwent extensive clinical evaluation and counseling before the MMA procedure. The Preservation MMA (PMMA) was performed following the strategy summarized in Fig. 1, and includes a preoperative, intraoperative and postoperative phase.

### Outcomes

Patient demographics included age, sex, race, and ethnicity. Race was categorized into White, Black, Asian, and Other. Ethnicity was reported separately from race and was dichotomized as Hispanic and non-Hispanic. Preoperative Epworth Sleepiness Scale (ESS) and Nasal Obstruction Symptom Evaluation Survey (NOSE) scores were collected [14, 15]. Preoperative PSG and DISE scores were recorded. SNOT-22 scores were collected preoperatively. The primary outcome was the change in SNOT-22 scores, one and three months after MMA. SNOT-22 scores were analyzed as a total score, total sinonasal score, which was the sum of questions one through twelve, and each symptom individually. The symptoms included under the total sinonasal score included need to blow nose, nasal blockage, sneezing, runny nose, cough, post-nasal discharge, thick nasal discharge, ear fullness, dizziness, ear pain, facial pain/pressure, and decreased sense of smell/taste. Postoperative ESS and NOSE scores were also collected at one month as secondary outcomes. Postoperative PSG variables were reported at least three months after MMA. Hypopneas were defined using the American Academy of Sleep Medicine (AASM) criteria using rule 1 A (3% or greater oxygen desaturation) or 1B (4% or greater oxygen desaturation) depending on the preoperative PSG scoring rubric. Postoperative hypopneas were reported consistently postoperatively to determine surgical success. Surgical success was defined based around the Sher criteria: final AHI reduction of 50% or greater [16]. Preoperative CT scans were also analyzed and a Lund-Mackey score was calculated using the following grading criteria: 0 for no abnormality, 1 for partial opacification, and 2 for complete opacification at the frontal, anterior ethmoid, posterior ethmoid, sphenoid, and maxillary sinuses, with the ostiomeatal complex being graded either as a 0 or 2. Biopsies of the sinonasal mucosa were also taken bilaterally at the time of surgery and sent for routine pathological analysis.

### Statistical analysis

Categorical variables were reported as frequency (N) and percentage (%). All continuous variables were analyzed for normality using the Kolmogorov-Smirnov test. Continuous variables were described as either mean ± standard deviation (SD) or median and interquartile range (IQR) when appropriate. Comparisons between preoperative SNOT-22 scores and postoperative SNOT-22 scores were performed with repeated measures ANOVAs. The sphericity of the data were tested using Mauchly’s test of sphericity. If data violated the assumption of sphericity, a Greenhouse-Geisser correction was used to calculate the *p*-value. Post hoc analyses were performed using a Bonferroni adjustment. Partial squared etas were calculated to compare the effect of MMA on the change of continuous variables postoperatively. According to published conventions, effect sizes were interpreted as small (0.01), moderate (0.06), and large (0.14) [17]. A *p*-value < 0.05 was considered significant for all statistical tests. All statistical analysis was performed using SPSS 27.0 (IBM Corp. Armonk, NY).

## Results

### Patient population

A total of 20 patients were included in the current study (Table 1). Being white (75.0%), and male (80.0%) were the most prevalent demographic factors. All subjects underwent maxilla-first surgery with patient specific implants. The mean Body Mass Index (BMI) of included patients was 34.4 ± 8.3 kg/m^2^ (Table 2). Patients who underwent MMA had a median ESS of 15.0 [6.3–17.8] and mean NOSE of 55.9 ± 28.4. The mean apnea-hypopnea index (AHI), respiratory depression index (RDI), and oxygen desaturation index (ODI) on preoperative PSG were 65.1 ± 28.9, 67.4 ± 32.9, and 75.1 ± 10.8, events per hour, respectively. The mean central apnea index (CAI) was 2.1 ± 5.3 events per hour. Oxygen saturation (SpO2) nadir median was 78.0% [69.0–82.0]. The median time less than SpO2 90% was 46.1 min [25.4–115.0]. All patients underwent septoplasty and bilateral maxillary antrostomy, with 35% of patients having maxillary sinonasal pathology present at the time of surgery. Lund-Mackey scores preoperatively was 2.8 ± 2.0.

**Table 1:** Demographics of patients who underwent MMA (*N* = 20)

Characteristic	*N* (%)
**Age at MMA**,** years (range)**	50.7 (31.0–65.0)
**Sex**	
Female	4 (20.0)
Male	16 (80.0)
**Race**	
White	15 (75.0)
Black	4 (20.0)
Asian	0 (0.0)
Other	1 (5.0)
**Ethnicity**	
Hispanic	0 (0.0)
Non-Hispanic	20 (100.0)

MMA = maxillomandibular advancement; N = number of patients

**Table 2:** Clinical outcomes of patient who underwent MMA (*N* = 20)

Characteristic	Preoperative	Postoperative	*P*-value
	Mean ± SD or Median [IQR]	Mean ± SD or Median [IQR]	
**BMI**	34.4 ± 8.3	32.2 [28.5–37.4]	
**PROM Scores (N = 20)**			
Epworth Sleepiness Scale	15.0 [6.3–17.8]	5.0 [2.8–7.3]	0.001**
Nasal Obstruction Symptom Evaluation	55.9 ± 28.4	8.1 ± 12.0	< 0.001***
**PSG** (*N*** = 12)**			
AHI, events/hour	65.1 ± 28.9	13.3 ± 11.7	< 0.001***
CAI, events/hour	2.1 ± 5.3	0.0 [0.0-0.3]	
RDI, events/hour	67.4 ± 32.9	20.1 ± 13.4	< 0.001***
ODI, events/hour	75.1 ± 10.8	6.1 [1.9–18.6]	< 0.001***
SpO2 Nadir	78.0 [69.0–82.0]	84.0 ± 4.1	0.003**
Time < 90%, minutes	46.1 [25.4–115.0]	0.5 [0.0-5.3]	0.039*
**Septoplasty**	20 (100.0%)		
**Maxillary Antrostomy**	20 (100.0%)		
**Maxillary Sinus Pathology**	7 (35.0%)		
**Lund-Mackey Score**	2.8 ± 2.0		
**Surgical Success**		12 (100.0%)	

MMA = maxillomandibular advancement; N = number of patients; SD = standard deviation; IQR = interquartile range; BMI = body mass index; PROM = patient reported outcome measure; PSG = polysomnogram; AHI = apnea-hypopnea index; CAI = central apnea index; RDI = respiratory depression index; ODI = oxygen desaturation index; SpO2 = oxygen saturation; *p < 0.05, **0.001 < *p* < 0.01, and ***p < 0.001

### SNOT-22 outcomes

Total score and total sinonasal scores significantly decreased postoperatively (49.0 ± 21.6 vs. 18.1 ± 17.4 vs. 12.5 ± 14.1, *p* < 0.001; 17.3 ± 12.5 vs. 9.2 ± 9.3 vs. 6.3 ± 7.3, *p* = 0.003) (Table 3). MMA had a large effect on total score improvement and total sinonasal score improvement (n_p_^2^ 0.72 and 0.35, respectively). Need to blow nose (1.9 ± 1.7 vs. 1.1 ± 1.3 vs. 0.7 ± 1.0, *p* = 0.026), nasal blockage (2.7 ± 1.7 vs. 0.9 ± 1.3 vs. 0.8 ± 1.0, *p* < 0.001), sneezing, (1.4 ± 1.5 vs. 0.6 ± 0.9 vs. 0.3 ± 0.8, *p* = 0.043), runny nose (1.4 ± 1.4 vs. 0.9 ± 1.0 vs. 0.5 ± 1.0, *p* = 0.038, cough (2.0 ± 1.7 vs. 0.4 ± 0.7 vs. 0.3 ± 0.8, *p* < 0.001), post-nasal discharge (1.3 ± 1.3 vs. 0.6 ± 0.8 vs. 0.3 ± 0.8, *p* = 0.002), dizziness (1.1 ± 1.3 vs. 0.6 ± 0.8 vs. 0.2 ± 0.4, *p* = 0.036), and ear pain (1.1 ± 1.6 vs. 0.6 ± 1.1 vs. 0.3 ± 0.5) were all significantly reduced postoperatively, with MMA having a large effect on each of these SNOT-22 variables. Facial pain/pressure was significantly worsened at one month postoperatively (1.2 ± 1.4 vs. 1.9 ± 1.5, *p* = 0.020), but then returned to baseline at three months (1.2 ± 1.4, *p* = 0.026). No significant changes in thick nasal discharge, ear fullness, or decreased sense of smell/taste were seen after MMA.

**Table 3:** Univariate Analysis of Effect of PMMA on SNOT-22 variables (*N* = 20)

SNOT-22 Variables	Pre	1 month	3 months	*P*-value	Partial Eta Squared
	Mean ± SD	Mean ± SD	Mean ± SD		
**Need to blow nose**	1.9 ± 1.7	1.1 ± 1.3	0.7 ± 1.0	0.026*	0.19
**Nasal Blockage**	2.7 ± 1.7	0.9 ± 1.3***	0.8 ± 1.0***	< 0.001***	0.53
**Sneezing**	1.4 ± 1.5	0.6 ± 0.9	0.3 ± 0.8	0.043*	0.20
**Runny nose**	1.4 ± 1.4	0.9 ± 1.0	0.5 ± 1.0*	0.038*	0.21
**Cough**	2.0 ± 1.7	0.4 ± 0.7**	0.3 ± 0.8**	< 0.001***	0.45
**Post-nasal discharge**	1.3 ± 1.3	0.6 ± 0.8*	0.3 ± 0.8*	0.002**	0.32
**Thick nasal discharge**	1.0 ± 1.3	0.6 ± 1.0	0.3 ± 0.08		0.14
**Ear fullness**	1.3 ± 1.6	0.8 ± 1.4	0.9 ± 1.3		0.07
**Dizziness**	1.1 ± 1.3	0.6 ± 0.8	0.2 ± 0.4**	0.004**	0.32
**Ear pain**	1.1 ± 1.6	0.6 ± 1.1	0.3 ± 0.5	0.036*	0.20
**Facial pain/pressure**	1.2 ± 1.4	1.9 ± 1.5**	1.2 ± 1.4*	0.026*	0.23
**Decreased Sense of Smell/Taste**	1.1 ± 1.4	0.5 ± 1.0	0.5 ± 1.0		0.11
**Difficulty falling asleep**	2.4 ± 2.2	0.9 ± 1.1*	0.6 ± 1.2**	< 0.001***	0.40
**Wake up at night**	3.6 ± 1.6	1.5 ± 1.7**	1.0 ± 1.3***	< 0.001***	0.60
**Lack of a good night’s sleep**	4.3 ± 1.3	1.1 ± 1.6***	0.9 ± 1.6***	< 0.001***	0.77
**Wake up tired**	4.3 ± 1.3	1.2 ± 1.6***	0.8 ± 1.4***	< 0.001***	0.76
**Fatigue**	4.0 ± 1.3	1.3 ± 1.7***	0.7 ± 1.1***	< 0.001***	0.74
**Reduced productivity**	3.6 ± 1.5	0.9 ± 1.3***	0.4 ± 0.09***	< 0.001***	0.72
**Reduced concentration**	3.4 ± 1.6	0.7 ± 1.2***	0.6 ± 0.9***	< 0.001***	0.66
**Frustrated/sad/irritable**	3.5 ± 1.6	0.9 ± 1.3***	0.7 ± 1.0***	< 0.001***	0.58
**Sad**	1.6 ± 1.8	0.6 ± 1.2**	0.4 ± 0.7*	< 0.001***	0.34
**Embarrassed**	1.3 ± 1.8	0.6 ± 1.1	0.4 ± 0.6		0.17
**Total Sinonasal**	17.3 ± 12.5	9.2 ± 9.3*	6.3 ± 7.3*	0.003**	0.35
**Total**	49.0 ± 21.6	18.1 ± 17.4***	12.5 ± 14.1***	< 0.001***	0.72

MMA = maxillomandibular advancement; N = number of patients; SD = standard deviation; CI = confidence interval; *p < 0.05, **0.001 < *p* < 0.01, and ***p < 0.001; * in 1month and 3 month columns refers to significance when compared to Pre values

MMA had a large effect on significantly reducing the majority of other SNOT-22 variables related to sleep, including wake up at night (3.6 ± 1.6 vs. 1.5 ± 1.7 vs. 1.0 ± 1.3, *p* < 0.001; 0.60). lack of a good night’s sleep (4.3 ± 1.3 vs. 1.1 ± 1.6 vs. 0.9 ± 1.6, *p* < 0.001; 0.77), wake up tired (4.3 ± 1.3 vs. 1.2 ± 1.6 vs. 0.8 ± 1.4, *p* < 0.001; 0.76), fatigue (4.0 ± 1.3 vs. 1.3 ± 1.7 vs. 0.7 ± 1.1, *p* < 0.001; 0.74), reduced productivity (3.6 ± 1.5 vs. 0.9 ± 1.3 vs. 0.4 ± 0.09, *p* < 0.001; 0.72), reduced concentration (3.4 ± 1.6 vs. 0.7 ± 1.2 vs., 0.6 ± 0.9, *p* < 0.001; 0.66), frustrated/restless/irritable (3.5 ± 1.6 vs. 0.9 ± 1.3 vs. 0.7 ± 1.0, *p* < 0.001; 0.58), and sad (1.6 ± 1.8 vs. 0.6 ± 1.2 vs. 0.4 ± 0.7, *p* < 0.001; 0.34).

### Postoperative outcomes

The BMI did not significantly decrease after MMA (32.2 [28.5–37.4] kg/m^2^, *p* = 0.959). ESS significantly decreased to a median of 5.0 (2.8–7.3, *p* = 0.001), and NOSE significantly decreased to a mean of 8.1 ± 12.0 (*p* < 0.001). Postoperative AHI, RDI, and ODI were all significantly reduced after MMA (13.3 ± 11.7, *p* < 0.001; 20.1 ± 13.4, *p* < 0.001; 6.1 [1.9–18.6], *p* < 0.001 events per hour, respectively). SpO2 nadir was also found to be significantly increased at 84.0 ± 4.1% (*p* = 0.003). Time in minutes less than 90% oxygen saturation was also significantly decreased to 0.5 min (0.0–5.3, *p* = 0.039). Surgical success by Sher-50 criteria (> 50% reduction) was determined to be 100.0%, and 75% had an AHI < 20. One patient showed a relapse at the sagittal split and was managed by refixation with successful stability for a year.

## Discussion

Maxillomandibular advancement is an effective surgical intervention for the treatment of persistent and/or severe OSA [1]. However, there is a concern in the literature that maxillary advancement can significantly compromise postoperative sinonasal function through a variety of mechanisms [2, 7, 9, 10]. Overall, there has been mixed evidence regarding the increased risk of nasal obstruction and rhinosinusitis after MMA or Le Fort I osteotomy specifically [2, 8, 9, 12, 18–20]. Therefore, this study was performed to further elucidate patients’ postoperative sinonasal function after performing PMMA with the sinonasal modifications published previously [2, 12]. Our study showed that both the total SNOT-22 and total sinonasal scores significantly improved postoperatively as early as one month postoperatively and persisted at three months postoperatively. The specific symptoms of the need to need to blow nose, nasal blockage, sneezing, runny nose, cough, post-nasal discharge, dizziness, and ear pain were all significantly reduced postoperatively. Facial pain/pressure did increase postoperatively at one month, but improved back to baseline at three months. These results suggest a potential improvement in sinonasal function after MMA when applying the forementioned sinonasal modifications.

In considering nasal breathing, our study showed that the subjective symptom of nasal blockage improved postoperatively after MMA. This is a significant finding because nasal breathing has been showed to be instrumental in treating OSA as it is involved with improving one’s quality of sleep and overall quality of life measures [2, 11]. This is supported by our prior findings using the Standardized Cosmesis and Health Nasal Outcome Survey, as well [12]. Furthermore, in patients with rhinosinusitis, nasal obstruction has been cited to be one of the most bothersome and common symptoms [21]. While it may be difficult to narrow down to a particular segment of sinonasal modifications as a direct cause for this improvement, the piriformplasty, inferior meatal antrostomy, nasal floor contouring, and transoral inferior septoplasty with inferior turbinate reduction, likely play a role in this symptom improvement [2]. Furthermore, as there is evidence that patients with OSA have decreased sinonasal quality of life and peak inspiratory flow, the PMMA inherently in itself may be a further contributor of improved sinonasal function [22].

**Fig. 2:**
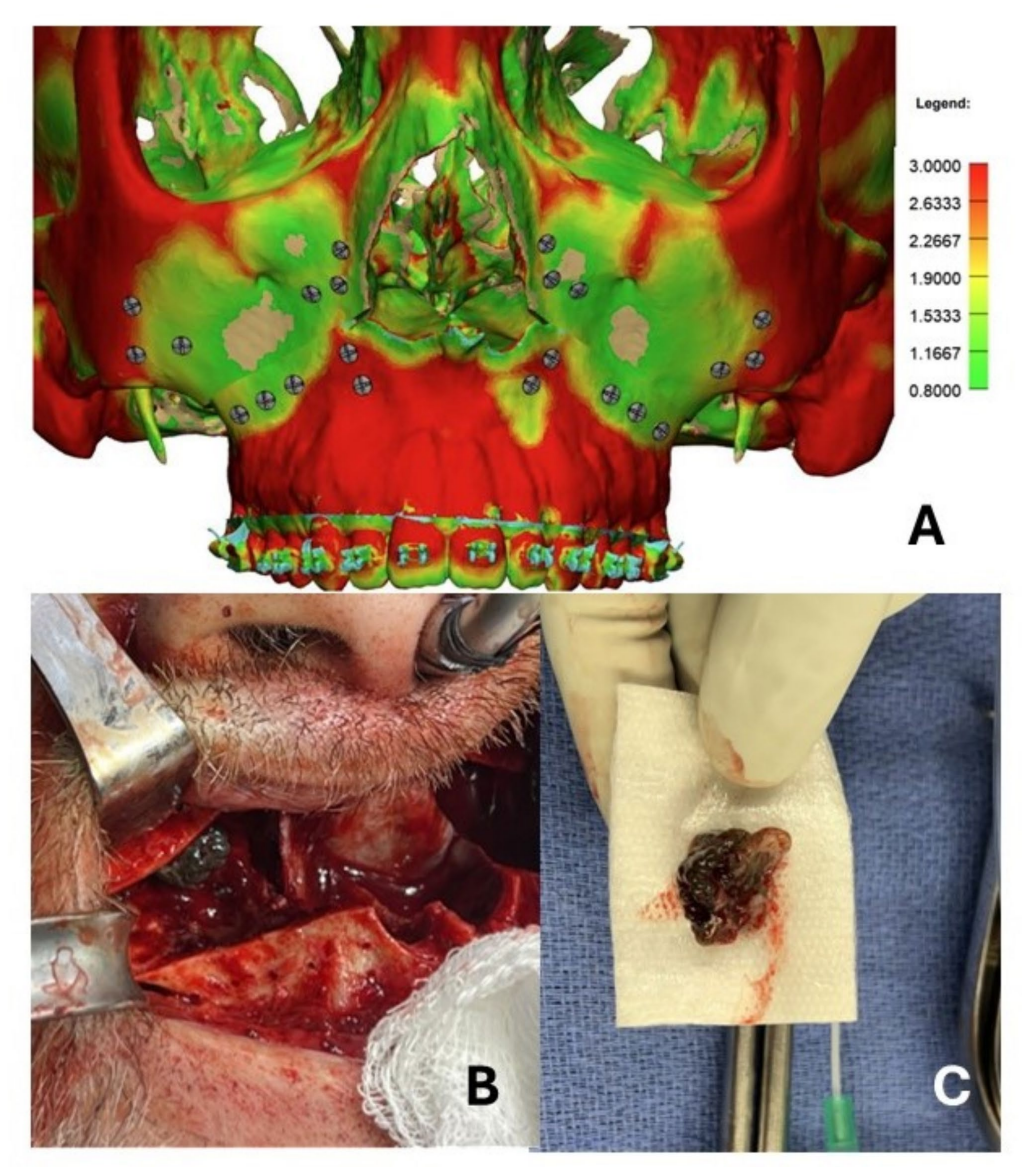
(**A**) VSP heat-map to assess maxillary bone thickness. (**B**) Right maxillary sinus after downfracture showing a persistent fungal ball after 3 endoscopic sinus procedures. (**C**) Fungal ball after extraction

**Fig. 3:**
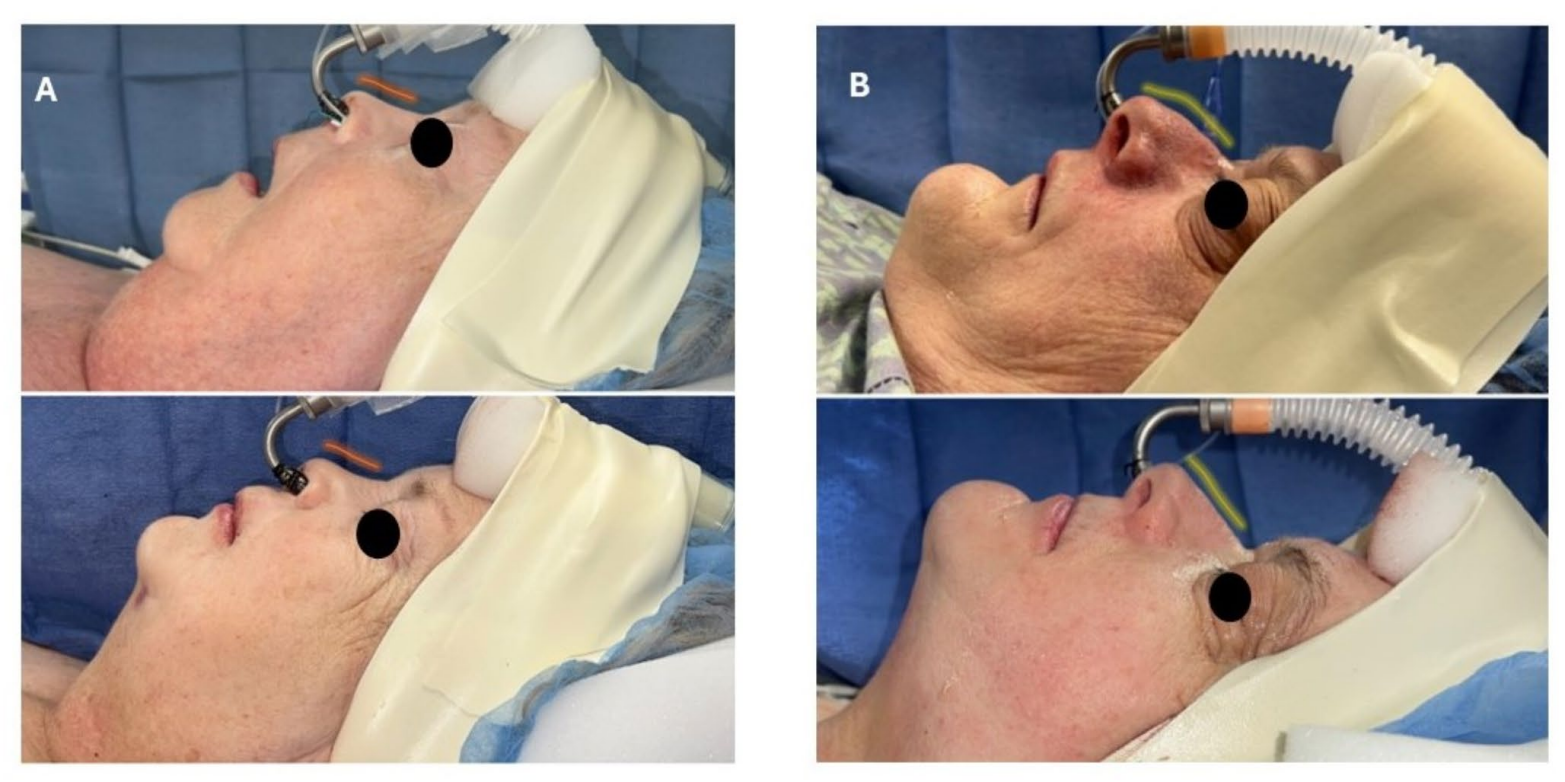
(**A**) Before and after of a patient with an over-rotated tip, septoplasty was performed to manage the septal deviation with incremental reduction of the caudal septal height to avoid addition tip rotation. Note the orange line unchanged. (**B**) Before and after of a patient who had a swinging-door technique to manage septal deviation and had the full caudal septum preserved to manage the ptotic nasal tip. Note the change in the yellow line

First, virtual surgical planning (VSP) has become increasingly common for a variety of orthognathic procedures, with emerging literature showing superior results when compared to traditional surgical planning [23]. In MMA for OSA, the advancements are not minor and require meticulous attention to the osteosynthesis technique. The VSP can also provide patient-specific implants which are critical in maxilla-first surgery. Another benefit is the heat-map provided to aid select sufficient bone thickness for allocating the prediction holes. Moreover, providing maxillary bone thickness can assist with screws length selection without compromising the maxillary mucosal lining (Fig. 2A). Also, since counterclockwise (CCW) rotation is usually applied in these severe OSA cases, attention to the length of the caudal septum is essential and should be determined on an individual basis (Fig. 3). Furthermore, inferior meatal antrostomy while preserving the maxillary sinonasal mucosa is applied where the medial wall of the maxillary sinus is drilled, with caution to preserve the descending palatine artery. Not only does this account for maxillary sinus drainage, but it also avoids deflection/blockage of the medial maxillary wall and reduces interference when CCW rotation or maxillary impaction is applied. Moreover, in some cases, a maxillary sinus pathology can be detected and managed after maxillary down-fracture, as shown in Fig. 2B-C. Lastly, septoplasty and inferior turbinate reduction have consistently been shown to improve nasal obstruction when indicated [24]; this study supports that performing these procedures in conjunction with MMA continues to have a positive effect on nasal breathing. While further research is needed to analyze if the improvement in nasal obstruction persists long-term, our study continues to support that MMA with these sinonasal modifications can improve sinonasal health.

Furthermore, in looking at the inflammatory response of the sinonasal cavity, patients after MMA reported a significant reduction in the need to blow nose, sneezing, runny nose, post-nasal discharge, and cough. In the orthognathic literature, there has been mixed evidence regarding the increased risk of sinusitis after a Le Fort I osteotomy, with some studies suggesting no increased risk of sinusitis [18, 19], some suggesting an increase in postoperative sinusitis by both symptomology and CT scan ratings [8, 9, 20], and others suggesting indeterminate effects on disease burden with the change in maxillary sinus size and function [25]. One study showed an increased incidence of chronic rhinosinusitis (CRS) to 7.2% after MMA, citing potentially changes to sinonasal mucosa or increased recirculation [26]. However, more research is needed to validate further if patients are at an increased risk of rhinosinusitis, especially in OSA patients. No other sinonasal symptoms were worsened after MMA. We emphasize preserving the integrity of the maxillary mucosal lining during the down-fracture, inferior meatal antrostomy and when selecting fixation screws length. Previous research has cited that disruptions in the mucosa integrity of the sinuses, disruption of mucociliary clearance, inflammation at the ostiomeatal complex, and increased in recirculation may all be playing a role in some reports of increased rhinosinusitis after MMA or Le Fort I osteotomy [8, 9, 20, 26]. However, other studies have shown that the maxillary mucosa heals appropriately after being violated, and while there may be more evidence of inflammation on CT scan, the correlation to symptomatic patient reported outcome measures (PROM) is minor [18, 19]. Interestingly, facial pain/pressure was worse at one month, but did improve to baseline at three months. It is difficult to know at this timeframe if this is secondary to changes in maxillary structure and potential sinusitis or if this is due to typical postoperative pain/care. While many informational guides on the internet endorse relatively mild pain after MMA, one study quantified postoperative pain and found that the majority of patients endorsed that the pain after surgery was tolerable [6], and another study found that MMA can be more tolerable than other sleep procedures, with one study showing that MMA had 50% less opioid consumption than palate surgery [27]. While further research needs to be done on the long-term effects on sinonasal function after MMA, this study supports the notion that rhinosinusitis may not be an early postoperative complication when the Le Forte is performed cautiously with the aforementioned sinonasal modifications. To our knowledge, this study is the first to highlight that patients can generally expect a relatively quick recovery of their sinonasal function and will likely not have to suffer with nasal symptoms for longer than a month after MMA.

While not the primary outcome of the study, it is worth noting the drastic improvement in subjective variables related to sleep quality, mood, productivity, and general quality-of-life. This aligns with other current research showing that other quality-of-life measures are markedly improved after MMA [28, 29]. Subjective PROMs are becoming more important in the study of OSA, as more research is showing the disconnection between objective OSA severity and individual subjective perceptions of OSA [30, 31]. Furthermore, patients with OSA endorse generalized quality-of-life and mood deficiencies rather than simply sleep quality issues, making the analyses of subjective PROMs even more important in further OSA research [32]. Interestingly, this phenomenon has been observed in chronic rhinosinusitis, where overarching reductions in cognition, productivity, mood, and other quality-of-life metrics were more pertinent to patients than the diagnostic criteria [33]. Ultimately, this study adds that MMA is an effective procedure for improving both the subjective sinonasal PROMs alongside the objective measures seen on PSG. Of note, MMA showed to almost eliminate the time spent below an oxygen saturation of 90%.

This study is not without limitations. Patients may develop symptoms at a longer follow-up as described in some previous works [26]. We hope to be able to continue this current study to follow the long-term effects after MMA as well as natural history on patients’ sinonasal function. That being said, due to the subjective improvement in patients’ sinonasal function even in the relatively early postoperative period with persistence to three months, it is necessary to report evidence on the impact of MMA on sinonasal function using validated PROMs, especially to properly provide evidence for clinicians and surgeons to effectively counsel patients considering MMA. Furthermore, this sample size was relatively small to stratify patients by their preoperative sinonasal exam findings. Patients can inherently present with different levels of nasal obstruction, internal valve collapse, inferior turbinate hypertrophy, and septal deviation. Future research with the addition of more patients who undergo MMA could focus on stratifying to see if a particular subset of patients does have worsening sinonasal function to aid in proper counseling and treatment. That being said, while we could use endoscopy or computed topography (CT) finding pre and postoperatively, sinonasal function is primarily determined by PROMs rather than objective measures, as these have been shown to be consistently poorly correlated [34]. Furthermore, it is difficult to ascertain whether the positive effects seen on sinonasal function are solely attributed to the sinonasal modifications, the PMMA, or to some combination of both. In the future, we may be able to compare our rates of sinus and septal pathologies after MMA to other studies to hopefully help ascertain the main driver of these improvements. Lastly, this study comes from a single population at a tertiary referral center in the Southeastern United States; therefore, our results may not be representative of all patients undergoing MMA, as regional differences has been shown amongst patients with CRS [35]. Potentially incorporating a multi-institution study using this protocol could further increase sample size and further improve diversity amongst our patient population.

## Conclusion

Patients who underwent PMMA with the sinonasal modifications for OSA did not show a compromise in their sinonasal functions, with some evidence that sinonasal function may improve after, particularly the need to blow nose, nasal obstruction, sneezing, runny nose, cough, and postnasal drip alongside improvements in all sleep-related items. Long-term follow-up with more patients is needed to support these findings.

## Data Availability

The datasets generated during and/or analyzed during the current study are not publicly available due to concerns of compromise of individual privacy, but are available from the corresponding author on reasonable request.
